# Understanding issues around use of oral pre exposure prophylaxis among female sex workers in India

**DOI:** 10.1186/s12879-021-06612-8

**Published:** 2021-09-08

**Authors:** Seema Sahay, Archana Verma, Suhas Shewale, Sampada Bangar, Athokpam Bijeshkumar, Mubashir Angolkar, Thilakavathi Subramanian, Nomita Chandhiok

**Affiliations:** 1 Division of Social and Behavioral Research, ICMR-National AIDS Research Institute, Pune, Maharashtra India; 2 Division of Epidemiology and Biostatistics, ICMR-National AIDS Research Institute, 73, G-Block, MIDC, Bhosari, Pune, Maharashtra 411026 India; 3 Department of Public Health, Jawaharlal Nehru Medical College, KLE University, Belagavi, Karnataka India; 4 Division of Social and Behavioral Research, ICMR-National Institute of Epidemiology, Chennai, Tamil Nadu India; 5 Division of Reproductive and Child Health, Indian Council of Medical Research, New Delhi, Delhi India

**Keywords:** FSW, Oral PrEP, India, Qualitative research, Violence, Stigma, Fear of side-effects, Alcohol use, Adherence, Reproductive health

## Abstract

**Background:**

Empowering female sex workers (FSWs) through women controlled HIV prevention option has been in focus globally. FSWs are important target for oral pre exposure prophylaxis (PrEP). A multi-centric qualitative study was conducted to explore the FSWs’ willingness to use oral PrEP in India.

**Methods:**

Seventy three interviews and 02 focus group discussions were conducted at 3 high HIV prevalent states in India during 2013–14. Study explored issues around willingness to use oral PrEP. The study was approved by the respective institutional ethics committee of the study sites. Thematic analysis using grounded theory approach was used to analyze the data in N-VIVO version 8.0.

**Results:**

Thematic analysis showed events of forced condom-less sex. FSWs believed that oral PrEP could provide independence, financial gains, and privacy and therefore hoped to use it as an alternative to male condom. However, any impact on physical/ aesthetic attributes and reproductive system were not acceptable and could become a barrier. Provider initiated oral PrEP was not preferred. Providers voiced safety monitoring concerns. Adherence emerged as a challenge because of: (1) alcohol use; (2) taking PrEP tablet each day being boring; (3) Stigma because Oral PrEP is ARV based. Alcohol use and dread of repetitive dose brings forth the need for long acting oral PrEP.

**Conclusion:**

Oral PrEP is acceptable among FSWs; it should be rolled out alongside strong messages on STI protection and PrEP as compliment to condoms. PrEP roll out requires educating communities about HIV treatment versus prevention. Long-acting oral PrEP could address both ‘boredom’ and alcoholism and sustain adherence.

## Background

The Indian epidemic is concentrated among vulnerable populations at high risk for HIV [1]. The concentrated epidemics are driven by unprotected sex between sex workers and their clients, men having sex with men and by injecting drug use with contaminated injections [2]. National AIDS Control Programme Phase-IV (2012–17) in India aimed to strengthen the response to the epidemic with key strategies of intensifying and consolidating prevention services with a focus on high-risk groups (HRGs) and vulnerable populations [3]. Female sex workers (FSWs) is the population that has shown response to the 'Combination HIV prevention program' that included behavioral change communication, barrier strategies (condom promotion), and structural programs [4] as evident from the declining trend in HIV prevalence between the year 2007 (5.06%) and 2011 [5]. Hence, FSWs might respond well to new HIV prevention options also. Therefore, empowering FSWs through women controlled HIV prevention options to reduce their risk of HIV infection has been a major focus of prevention efforts globally. Several clinical trials reported protective effects of biomedical interventions in the form of gel and oral formulations of pre-exposure prophylaxis (PrEP) but some trials were also stopped because they lacked efficacy [6–8]. Subsequent studies showed that lower efficacy was related to suboptimal adherence to the PrEP [9, 10]. Therefore, the dose–response relationship between efficacy and adherence to PrEP has received critical focus among researchers because of the subjective world view of adherence taking precedence over objective world view. An enhanced understanding of social and behavioral influences on PrEP use has been recommended [11, 12].

UNAIDS and the World Health Organization recommend PrEP as an additional prevention choice for people at substantial risk of HIV exposure and those who are ready to have regular HIV testing. However, a gap between efficacy and effectiveness is foreseen especially as clinical trial researchers observed adherence as a major challenge.

Adherence to preventive strategies is challenging because there is often no direct effect such as the reduction in any kind of ‘symptoms’ or getting a feeling of wellness. Acceptance and adherence are shaped within the socio-cultural, psychological and programmatic context in any geographical setting. Despite the strong biological effectiveness of oral PrEP owing to strong adherence, FSWs face many structural challenges to PrEP uptake and use [13]. Social scientists have raised the need to assess the subjective world view and social meanings of PrEP. They have warned to take cognizance of the perception of safety, trust, and empowerment [14]. A PrEP demonstration project was conducted among FSWs in Kolkata, India which has shown that there is a demand for PrEP among FSWs but socio-structural barriers influence PrEP uptake [15]. Studies on needs and perception of FSWs about PrEP uptake and its delivery is a gap in India. According to Bandura (1986), the human function is influenced by one’s characteristics; environment or context and behavior [16]. To understand the basic human function of decision making to use a preventive product in environmental, behavioral and personal context; a qualitative study was undertaken to get an emic perspective for oral PrEP uptake among the FSWs in India. Parkin’s theory of decision making process of problem definition; thought; judgment; decision; and action [17] was utilized to interpret the data.

## Methods

### Study setting

Based on the NACO’s categorization of districts as per HIV prevalence, two districts in each state which were identified as ‘category A’ (> 1% ANC prevalence in the district in any of the sites in the last 3 years) and ‘category B’ (< 1% ANC prevalence in all the sites during last 3 years with > 5% prevalence in any HRG site) [3] were selected as study sites. Using this criterion, Pune (urban) and Satara (rural) districts in the state of Maharashtra; Belagavi (urban) and Hubli-Dharwad (rural) districts in the state of Karnataka; and Chennai (urban) and Vellore (rural) districts in the state of Tamil Nadu were selected as study sites. The participants were categorized into 3 components: (1) Potential PrEP Users: FSWs, (2) Key informants: Brothel owners/ keepers, and (3) PrEP Providers: Health care providers.

### Sampling and study population

Purposive and convenience sampling techniques were used to recruit the participants and face to face interviews or group discussions were conducted. The participants were identified and recruited through Non-Governmental Organizations (NGOs), Community Based Organizations (CBOs) and key community gatekeepers such as tea stall owner/ worker, paan waala (the betel shop owner), small shop keepers and pimps. A total of 39 in-depth interviews (IDIs) were conducted with FSWs as follows: (1) Brothel Based Sex Workers (BBSWs) (n = 20), and (2) Street Based Sex Workers (SBSWs) (n = 19). Thirty-four key informant interviews (KIIs) were also conducted with six brothel keepers (BKs), one bisexual man and 27 health care providers/program personnel. Two focus group discussions (FGDs) were conducted with a total of 14 FSWs at two urban sites of Pune and Chennai respectively. BBSWs and BKs were approached through NGOs/ CBOs only. Recruiting SBSWs was a challenge. SBSWs were approached either through NGOs or an additional strategy of snowball technique was used. In qualitative research, sampling can occur at several stages, both while collecting data and while interpreting and reporting on it. The key is purposive or strategic sampling. Each SBSW whom we interviewed was requested to refer participant/s who would have information of our interest. The participants were self-identified as BBSWs, SBSWs, and BKs. The HIV status of the participants was not explored in the study because we exclusively wanted to gain community’s insights into HIV prevention methods. This was done to avoid any confusions in the community between ART based oral PrEP and treatment using Anti-retrovirals. Therefore, we did not explore the experience of being HIV positive which would have included treatment, stigma, access etc. The health care providers included physicians, obstetric gynecologists who were identified from government and private health centers, counselors from ICTC and NGO/CBO, and representatives from District AIDS Prevention and Control Society (DAPCU). Interviews were conducted at a confidential and convenient place agreed on by the participant. Each interview took 40–90 min. Four FSWs refused to participate in the study.

To provide a background understanding of the subject, the participants received information on recent research findings of oral PrEP before conducting the interviews/ FGDs. A description of oral PrEP was used as a preamble before the interview section about oral PrEP.

### Study tools and data collection

The interview guides for IDI and FGD focused on the perceived need for HIV prevention, attitudes and practices about family planning methods; knowledge and experiences of condom use for family planning and/ or prevention; knowledge, perception about STI and HIV/ AIDS, prevention methods, and usage modalities for oral PrEP. The study explored the opinion and expectations of the FSWs from oral PrEP, barriers and facilitators in using oral PrEP, and willingness to use oral PrEP. The guides were translated into local vernacular languages (Hindi, Marathi, Kannada, Tamil, and Telugu) by the study sites. The guides were pilot tested at all study sites and refined based on pilot findings.

All the interviews were conducted by trained master’s level female social workers in the local language in Maharashtra and Tamil Nadu. Data from Karnataka was collected by a trained female social worker and MPH female students. Field notes were taken during the IDIs and FGDs. The data were audio-recorded and data collection was continued until theoretical saturation was reached. As fieldwork progressed, data was continuously analyzed using constant comparison and iterative thematic coding approach [18]. The reimbursement of Rs. 150 was given to all study participants.

### Data analysis

The audio data was transcribed verbatim, translated into English and typed in Microsoft Word at the study sites. The processed translated electronic data were repeatedly reviewed by two researchers and the PI. Repeat interviews were requested from the sites in case of missing information or if there was a need for new information. The repeated sections underwent a similar process of data processing and finalized data was entered in qualitative software N-VIVO version 8.0. Attributes were tabulated in the software to quantify demographic variables of location, typology, age, and marital status. Data were coded to recognize similarities and differences using the constant comparison method [19]. Firstly, the themes were identified deductively from the interview guide and subsequently inductively from the data. Following the iterative process of reading, final themes emerged using the grounded theory approach [20]. Data were coded by two researchers SS and AV, first independently and then discussed together. The codes and the descriptors were shared with the site PIs for confirmation and their interpretations were included to preserve the local meanings. The coded data were analyzed which informed the next iteration of data collection until a strong theoretical understanding was attained and these are described as emerging themes.

### Ethics

The study was approved by the Institutional Ethics Committees of all the 3 sites: the ICMR-National AIDS Research Institute (Pune, Maharashtra), the ICMR-National Institute of Epidemiology (Chennai, Tamil Nadu) and the Jawaharlal Nehru Medical College (Belagavi, Karnataka). Written informed consent was obtained from all the study participants for participation in the study and audio recording of their responses before conducting the IDIs, KIIs, and FGDs.

## Results

Of the total 59 FSW participants, 42 belonged to the urban setting while the rest belonged to the rural setting. The mean age of the FSWs in both urban and rural settings was similar (35.7 years, SD = 8.8) (Table 1). The qualitative data was analyzed thematically to capture willingness to use oral PrEP and understand the dynamics of the various factors that affect the usage, acceptance, and adherence of PrEP among a high-risk HIV group, the FSWs, in various parts of India.

**Table 1:** Demographic profile of study participants (female sex workers)

	Brothel-based sex workers (n = 20)	Street-based sex workers (n = 19)
Characteristics		
Age range (mean age)	22–50 (35.2)	25–63 (36.8)
Education		
Illiterate	42% (08/19)	22% (04/18)
Up to primary	21% (04/19)	28% (05/18)
Up to 10th	37% (07/19)	50% (09/18)
Missing	01	01
Occupation		
Sex work only	13 (65%)	11 (58%)
Sex work and working with NGO	04 (20%)	06 (32%)
Sex work and housewife	02 (10%)	01 (5%)
Sex work and other work	01 (5%)	01 (5%)
Marital status		
Unmarried	02 (10%)	03 (16%)
Married	08 (40%)	09 (47%)
Separated/ divorced	04 (20%)	02 (11%)
Widowed	04 (20%)	03 (16%)
Live-in	02 (10%)	01 (5%)
Type of family		
Nuclear	10 (50%)	08 (42%)
Joint	03 (15%)	03 (16%)
Living alone	07 (35%)	08 (42%)

**Fig. 1:**
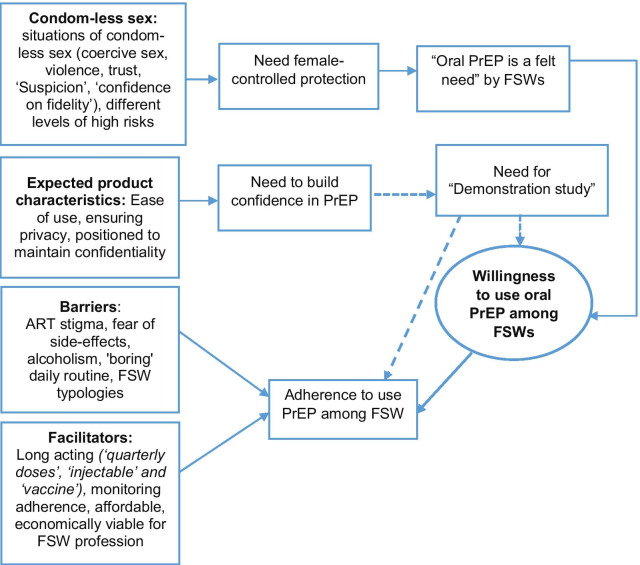
Analytical framework for emerging needs for oral PrEP use among FSWs. The need for PrEP emerged because FSWs were constantly at risk even if they were aware of condom and wanted to use condom. Vulnerability narrated by FSWs brought forth the 'need’ for oral PrEP. Demand for demonstration study emerged as influencer of usage but there were other facilitators that could influence usage independent of demonstration studies. Long action products would help in future adherence. The dotted lines indicate desire for demonstration project or need for contextual evidence

All the FSWs from both the settings reported experiencing coercive sex which involuntarily brought out the risk of condom-less sex. Several other situations also led to condom-less sex. The main emerging theme in this study was ‘condom-less sex’ which led to the theme of ‘oral PrEP is a felt need’. The latter encompassed issues such as female-controlled options, an alternative to male condom, long-term protection, easy to use, and economically viable option. As PrEP is not rolled out in India, respondents expressed the testing of PrEP products for safety and feasibility before their actual usage. Therefore, 'building confidence in product' was another emerging theme for acceptance. Influencers for PrEP acceptance and usage such as ease of use, and privacy emerged from the data while stigma and fear of side-effects emerged as barriers for PrEP usage among the FSW population. However, the reiterative readings helped in identifying the characteristics and issues that would lead to acceptance of oral PrEP under the theme 'desired product characteristics' (Fig. 1). This theme covered the issues of ease of use, ensuring privacy and positioning PrEP in a way to maintain confidentiality. Experiences of antiretroviral therapy and their job profile threw light on both acceptance and adherence issues. ‘Adherence’ was another theme in the study.

The major themes that emerged from the study were: (1) condom-less sex, (2) oral PrEP is a felt need, (3) building confidence in the product, (4) desired product characteristics, (5) barriers to acceptance, and (6) opinions about adherence to oral PrEP.

### Context of condom-less sex

While explaining their work, FSWs shared about their vulnerabilities. Commenting on the lack of protection policy for their population, they narrated incidences of coercive sex and situations where they would end up having condom-less sex. FGD participants unanimously reported that like any other woman who have not been in sex work, they too faced resistance to condom use in regular partner settings. This is important to understand that condom is male oriented prevention method and women either FSW or Non FSW, face similar resistance from their regular partners for its use for various reasons. Since, this resistance is similar in women who are at low and high risk of HIV acquisition, there is need for women oriented prevention options. ‘Suspicion’ because of condom use; an emphatic ‘no’ to condom use by the partner; ‘confidence on fidelity’—were the phrases used by FSWs to explain condom-less sex in certain types of relationships. FSWs stressed that livelihood needs itself brought vulnerability. An SBSW from Pune narrated the situation where being aware of the risk did not necessarily result in the practice of using a condom:

“Now I came to you for work [/maid work-FSWs reported various occupations also/] but that owner likes me. If the female owner went outside, then, he takes that maid servant to the bed [for sex]. Is there a condom [available at that time]?” [Urban SBSW, 01-10-13, Pune, MH]

The universal nature of the risk was apparent at all the settings. Violence and getting caught in the exploiting situations where FSWs were forced to provide services to multiple people, were reported at all the sites in both rural and urban areas. An FGD participant from the another city reported:

“Some person [/client/] will call us and ask us to have sex in the nearby bushes. When we go, we see there, then there will be 6 to 7 people. It is very much difficult to handle the situation, and cannot use a condom with all of them!” [Urban FGD, FSW-4, Chennai, TN].

In rural Karnataka (Hubli district), FSWs talked of fear of getting killed if they resisted:

“What to do? We don’t have any options. They [/clients/] come and tell that there is only one person, but they will be four and they will take [us] to the jungle and will do [sex] forcefully. If we go against them, they will kill us and will throw [us] there only” [Rural BBSW, 03-10-35, Hubli-Dharwad, KT].

Instead of didactic education, practical need for protection emerged. Need for protection was urgent because of fear even from law enforcers. A lodge-based FSW from Belagavi explained as follows:

“Government should give us more facility [/safe space/] that also in free. We are poor. So sometimes police and ‘don’ [/local goons/] people come and take [us] forcefully [for sex] and put us in jail, and then they are asking for money and doing nonsense things [/having sex/] with us. Then madam [/ Brothel Keeper/] goes from here to bring us out from jail. We are in serious trouble in Belagavi”. [Urban BBSW, 03-10-35, Belagavi, KT]

FSWs did not use condom with their regular partner for fear of suspicion and violence when condom use was suggested:

“We cannot insist upon all [/types of partners/] to use condom. If we insist on a husband or partner to use a condom, then they will suspect us and violence might happen” [Urban FGD, FSW-2, Chennai, TN].

The emotional need of getting accepted by the regular partner led to condom-less sex even among the empowered peers. ‘Trust’ in a relationship turned out to be a barrier to condom use:

“My partner has seen my report. I don’t have HIV [infection]. [He] has seen my report, and he sits [/has sex/] with me without a condom. What to do?” [Urban SBSW, 01-10-13, Pune, MH]

In the setting of stigma and societal facelessness, trust shown by her partner is a valued reflection of respect given by the partner which she would not want to lose at any cost. To reciprocate, she does away with condom use. An FSW in Chennai stated:

“I don’t have a husband but I have one regular partner. And he has a lot of confidence in me. So due to this high confidence in me, I never use a condom with him” [Urban FGD, FSW-4, Chennai, TN]

### Oral PrEP is a felt need

Despite being aware of risks and working in the fear of situations of coercive sex, FSWs did not refuse to go ‘outside’ with clients. Condom-less sex events seemed to be a common occurrence. Varying levels of risk brought out the need for FSW controlled prevention option. An FSW shared her need for oral PrEP as follows:

“Sometimes if we get caught in the custody of rowdies, they threaten us and ask us to have sex without using a condom. So because of fear, we have sex without a condom with them. To prevent HIV transmission, it [/oral PrEP/] is needed, like earlier sister said that, at the time of alcohol consumption they do sex without a condom” [Urban FGD, FSW-1, Chennai, TN].

In the context of condom-less sex, oral PrEP as an alternative to male condom was a felt need of both rural and urban FSWs:

“Definitely, the new prevention product is very much necessary because the gents [/men/] refuse to use the condom during sex. This new prevention product will be very much necessary for people like us [/FSWs/]” [Rural BBSW, 06-10-44, Vellore, KN].

Oral PrEP was thought as addressing following issues: Pleasure to the clients; protection and financially beneficial at individual level of FSW's profession.

*Pleasure for clients:* Need for oral PrEP, especially as a female-controlled option, was acceptable to both the brothel (42.7%) and street-based (57%) sex workers. They reported facing challenges around male condom usage such as ‘interference with pleasure’ and ‘client satisfaction’. A female key informant from an NGO emphasized upon the client’s expectation of pleasure and how oral PrEP would satisfy that criteria:

“It [/oral PrEP/] is convenient to take! There is no question. People feel that there is no skin to skin touch in condom use. That issue doesn’t come in that [/when one uses oral PrEP/]” [Urban Social worker, 01-60-16, Pune, MH].

FSWs in the FGD agreed as follows:

“Some people say that they feel dissatisfaction during sex [/with condom/]” [Urban FGD, FSW-5, Chennai, TN].

*More controlled protection:* Violence and forced sex were the social conditions in which an FSW lived. Within this social context, an FSW who is aware of the threats but her livelihood needs make her take a risk, depicts the multi-layered nature of her vulnerabilities. Transactional sex is ‘hurried’ which leads to condom tear. These situations give rise to the need for other prevention technologies:

“Some customers [/client/] will tear the condom and do [sex]. In such times, we should be careful. So this tablet [/PrEP/] will be useful in that condition” [Urban SBSW, 05-10-19, Chennai, TN].

Since client satisfaction is the major goal in the sex work profession, a health care provider (HCP) from a rural site emphasized PrEP as an empowerment option for FSWs.

“If it [/PrEP/] is to be taken regularly, target intervention people especially female sex worker can take it. Because they can use it when the male condom is not used. A female condom is not there [/available/]” [Rural DAPCU representative, 06-50-41, Vellore, KN].

*Perceived as profitable:* Condom-less sex being more in demand, FSWs participating in FGD had hoped for an alternative for the male condom. They examined its economic benefit along with its long term protection value:

“Some people are there if the clients give more money then, they have sex without using a condom” [Urban FGD, FSW-2, Chennai, TN].

She emphasized her need for long term protection which a tablet formulation would be able to give her along with non negotiable sex work with clients who do not want condom. The empowerment due to PrEP tablet reflected as follows:

“...It [/Prevention option/] must come in the form of a tablet so that it will be in our body and we can do sex work without any problem. Eventually, we can earn more money and also we can live safely” [Urban FGD, FSW-2, Chennai, TN].

Although no one talked about pregnancy prevention or STI prevention they were aware about use of condom for prevention from STIs.

##FC## CAN WE USE CONDOM AFTER DOING FAMILY PLANNING OPERATION?

##FSW 1–8## Yes, it is must. [/Chorus answered by the respondent /]

##FC## WHAT THEY WILL THINK OF USING CONDOM BY THE RISK AND WITHOUT RISK PEOPLE?

##FSW-2## Those who are at risk and without risk, they use different and easy method, they must use condom regularly if they attend one or ten clients for their safety, at the same time they must use one condom for each client.

##FSW-5## It is a safety and good thing.

### Building FSWs confidence in the product

A high level of acceptance for hypothetical oral PrEP was expressed among FSWs belonging to the urban areas of Chennai (10/ 13) and Pune (03/ 05). However, the need to be confident about the actual product prevailed:

“Now, without seeing how we can tell [about] those things? [/she laughs/] Without seeing, how can the things be known? As we saw [/used/] the condom and accepted… so will check the thing [/oral PrEP/] after it comes and then only will prefer” [Urban SBSW, 01-10-13, Pune, MH].

“It is very much difficult to take the tablet regularly. Before taking this tablet [/PrEP/] regularly, we must ‘know’ about the tablet [/PrEP/]” [Urban SBSW, 05-10-77, Chennai, TN].

Participants felt they need to use a product to know the range of side effects it may cause and it might influence the sustained use of this prevention product:

“Only after using it, we can tell whether it has any problem. But it should not have side effects.” [Urban SBSW, 05-10-19, Chennai, TN].

In Chennai, participants were conscious of the need for the product to be tested first:

“Before launching the tablet, the tablet must be pre-tested. Then only people will come forward to buy and use” [Urban FGD, FSW-3, Chennai, TN].

### Desired product characteristics

If FSWs were confident about the product and wanted to use it, they had suggestions for certain characteristics of the product which would facilitate optimal usage of the product. Following three facilitators emerged from the study: (1) easy to  use, (2) ensuring privacy-formulation, (3) positioning to maintain confidentiality.

*Ease of use*: Among many of the challenges with condom usage, making men wear a male condom was reported to be the major challenge for FSWs. They found the process of wearing a condom far tedious which in their own words was ‘not as simple as swallowing a tablet’.

“This tablet is for preventing HIV. So, it can be very well taken. We don’t have a fear of it. Whoever wants, can use it. Men or women, whoever is going to do this type of work [/sex work/], they can use it. It is good to use it as it prevents HIV” [Urban BBSW, 05-10-27, Chennai, TN].

*Formulation to ensure privacy desired:* FSWs wanted to protect their privacy; a client need not know if she was using any protection. This privacy also appeared to be a function of ‘trust’. Without invading the ‘trust’ of the partner, the FSW would be able to use the pill to protect herself. Swallowing a pill would give her protection, privacy, and ease of use, altogether. According to them, since it would be a pill, it would be easy to use:

“Among all, tablets are best madam. Means it will be swallowed if that tablet is taken- means- no one will come to know” [Rural SBSW, 04-10-08, Dharwad, KN].

The ‘oral tablet’ allayed FSWs’ fears about their confidentiality; they felt that it made them self-sufficient for protection; did not make them dependent on the health system:

“Tablet is the best. We don’t have to go to a place for injection, we can buy and keep the tablets and use by ourselves. Even this has to be made available in NGOs, medical shops and hospitals” [Urban SBSW, 05-10-19, Chennai, TN].

There seemed to be no realization that oral PrEP might not be an ‘over the counter’ (OTC) drug and that there would be a need for monitoring by the HCP for any side effects after the initiation of oral PrEP. FSWs from Chennai during FGD pointed out their preference for CBOs or NGOs and not doctors for oral PrEP dispensing as they wanted to be given services ‘with no questions asked’.

“Now the doctor asks many questions - how many customers I have attended? Do you have a habit of alcohol? How much money do you spend on that?” [Urban FGD, FSW-7, Chennai, TN]

“This may be the reason people are not coming to the hospitals because unwanted questions were being asked by the doctors... so this may be the reason that people hesitate to come to the hospital and ICTC centers” [FSW-2].

*Positioning as medical product:* The theme ‘Positioning to maintain confidentiality’ was derived from the narratives where FSWs felt that they would be able to access the tablet in privacy; where no one would know them and no one would understand the purpose. According to them, the positioning of the product as a medical product would be helpful. If the product is sold as medical product without attaching stigma of high risk behavior, it would be acceptable and accessed:

“It will be good if it comes in medical products like a tablet, so that without anyone knowing [about it], we can have the tablet” [Rural BBSW, 06-10-44, Vellore, TN].

“…normally anyone goes to medical [/pharmacy/]” [Urban SBSW, 01-10-13, Pune, MH].

Unlike FSWs in an urban setting who did not want to go to a doctor to receive oral PrEP, FSWs in rural areas did not have any concern about the integration of oral PrEP delivery with ART centers or ICTCs. Their idea was to integrate CBOs/ NGOs with existing program structures:

“From the ART center, it should be promoted up to the outside people again… it is very necessary [to be dispensed], through an organization. From there also the women will get information” [Rural BK, 02-60-44, Karad, MH].

“I feel that… somewhere if it [/tablet/] will be kept in NGOs then will be ok. I do feel so” [Rural SBSW, 02-10-42, Karad, MH].

From the implementer’s point of view also, dispensing through village level functionaries was the preferred option:

“Availability is very important for the tablet. If it is available with village health nurses, Anganwadi workers [/village level workers/] it will be still good” [Rural DAPCU representative,06-50-41, Vellore, KT].

### Barriers to acceptance

Many concerns about oral PrEP were expressed. Taking a tablet for prevention is a major issue. Following barriers to oral PrEP usage emerged: (1) Stigma of using HIV treatment product, (2) Fear of side effects.

*Stigma of using HIV treatment (ART) product:* FSWs were apprehensive about accessing oral PrEP tablets because they felt that it would be equated to seeking treatment for HIV by the community. They used phrases such as, ‘becoming infamous [/defamed/]’; ‘community would never believe [/that they are uninfected/]’. Despite the emerging need for oral PrEP, FSWs cautioned that oral PrEP might become stigmatizing. After all, the Oral PrEP is an *‘*ARV’. They were conscious of the fact that the community perceived oral PrEP tablets as a ‘treatment medicine’ for ‘the HIV infected’. To differentiate between oral PrEP and ARV was too complex to discern for them and the community:

“No one will agree for that tablet [/oral PrEP/]. So women will be afraid of becoming infamous. =name of FSW= as well as =name of another FSW= eats the same tablets! It means they both have got [/HIV/]. There the people [/client/] will say this who is [/point out/] having the same tablets. That tablet [/ARV/] has become too famous” [Urban SBSW, 01-10-13, Pune, MH].

A woman mimicked the voice of the community:

“This means the tablets cannot be taken without a reason. I don’t have any disease, nor any risk. Why then I eat [/tablet/]?” [Urban Woman (Spouse of PLHIV), 01-20-23, Pune, MH]

*Fear of side effects: *In addition to the above described reasons for non-use of oral PrEP, fear of side effects was another major cited reason. Most of the BBSWs (77.3%) talked about their concerns about side effects. However, SBSWs raised this concern in low numbers (5.6%).

“… But for some people, the body will not accept the drug. Sometimes they may take the tablet and some time they may skip” [Urban BBSW, 05-10-27, Chennai, TN].

‘Body heat, giddiness, ulcers, weakness and total rejection by the body’ were some of the anticipated side effects of oral PrEP. Fear of side effect was also voiced as reasons for preferring condoms over oral PrEP.

“I don’t know whether people will use regularly. Those who like to use condom definitely, they may have fear on using this tablet [/PrEP/] so there will be less chance of using this tablet [/PrEP/]” [Urban BBSW, 05-10-72, Chennai, TN].

FSWs professed fear towards the possibility of harm occurring to their reproductive parts. They were quite vocal about this issue and some narrated their experience from family planning.

“But I feel difficult to use regularly because earlier I used =mala-d= [/contraceptive pills/] that gives many problems like a bad smell in urine, lower abdomen pain and burning sensation” [Urban BBSW, 05-10-72, Chennai, TN].

Related to their profession, preserving the beauty and the reproductive part/s, were some of the major concerns. In both urban and rural sites, concern existed among the FSWs that these attributes might get compromised if they took oral PrEP:

“People will take regularly to prevent diseases. This tablet we can take, but, there should not be any problem in the uterus of the women” [Rural BBSW, 06-10-44, Vellore, TN].

“Only if it is not having side effects, we can use it regularly. Beauty is a must to do sex work. This tablet should not spoil it” [Urban SBSW, 05-10-19, Chennai, TN].

### Adherence to oral PrEP: perceived barriers

Despite the proven efficacy of oral PrEP, the effectiveness depends on the adherence. The daily dose was a challenge:

“FSW people… Taking the tablet daily is impossible for them. Other products may be used by others” [Urban BBSW, 05-10-30, Chennai, TN].

Three sub-themes emerged that cover the perceived barriers to adherence to oral PrEP: (1) alcoholism, (2) psychological barriers, (3) typological barriers. These barriers to adherence are discussed as follows:

*Alcoholism: *Alcohol consumption is a compulsion of her profession and subsequently, it becomes her need. Hence a vicious cycle of compulsion and alcoholism appears to be the lifestyle under which FSWs showed apprehension for initiating or adherence to oral PrEP tablets. According to an FSW in Chennai:

“Sex workers will maximum [/mostly/] be alcoholics. They say that only when they are drunk; they can do the work. We don’t know whether this tablet can be taken if drunk? Suppose if it can be used even in drunken [state], then again taking it regularly will be difficult because if they take alcohol, they forget to take tablet regularly...These people [/alcoholics/] are not taking tablets regularly even for diabetes” [Urban BBSW, 05-10-30, Chennai, TN].

SBSW from Chennai pointed out towards the habit of consuming alcohol and raised apprehensions about interactions between alcohol and oral PrEP:

“Most of the people in the field [/sex work/], they are having the habit of alcohol consumption. I have a doubt! While using this tablet, how it works at the time of alcohol intake?” [Urban SBSW, 05-10-21, Chennai, TN].

Alcohol was professed as part of their lifestyle. An SBSW from Pune described how she ends up having alcohol in her occupation and that she would be anxious about interaction with PrEP:

“Then my partner, if we have gone outside then what he does, at very first he brings beer, biryani [/a rice-based spicy and savory food/], etc. If with him, then now…after taking the tablet, if I took a beer with him, then, will I not have trouble [/Taking beer with PrEP tablet: Would adverse effect develop? /]” [Urban SBSW, 01-10-60, Pune, MH]

In an FGD at Chennai, an FSW also wanted to know about interactions with recreational drugs. One of the FSWs in the FGD also logically pointed out that because daily alcohol or drug intake was the compulsive need of their profession, taking daily oral PrEP will be a challenge:

“At the same time, I don’t know whether those who are addicted to drugs they might take or not [/take oral PrEP/]. They feel that they would not be able to work if they are ‘not intoxicated” [FSW-8].

A complete conviction seemed to emerge that this tablet should not be taken with alcohol or any other substance as the FGD participants from Pune put forth this argument:

“If having taken the tablet, then we should not chew Gutkha [/chewable tobacco/], should not drink [alcohol] and I have seen that with my eyes” [FSW-4].

Alcohol and drug use were the strong perceived reasons for not taking PrEP inadvertently: ‘they will forget’; ‘will be too intoxicated to remember’ were commonly uttered phrases in the study. An SBSW from Pune told the reason for not wanting to take PrEP:

“Yes, hurdles… therefore we can’t take [oral PrEP] daily and one thing about me is, I have the habit of drinking [alcohol]” [Urban SBSW, 01-10-60, Pune, MH].

The apprehensions surrounding PrEP medicine and alcohol was prevalent among FSWs. An SBSW from Pune narrated the sequelae of ending up with consuming alcohol for any or everything:

“As I drink alcohol, it means I cannot take that tablet [/Feels PrEP would interact with alcohol/]. Suppose I have white discharge then also I will not take that tablet; I will take it on the second day. But then, as I have severe white discharge; I will become angry and drink one quarter [/of alcohol/]. Women [/FSWs/] have such [habit] and [only] 5 out of 100 women [/FSWs/] are there who do not drink!” [Urban SBSW, 01-10-13, Pune, MH].

Thus, fear of the adverse effect of oral PrEP in the context of their lifestyle was a major concern.

*Psychological barriers: *A brief attention span and sustaining interest emerged as a unique challenge among FSWs. The fluctuating mood is aptly reflected in the phrase they used: ‘anything routine is boring’. They do not want a repetition of any nature in their life and therefore, taking medicine every day is ‘boring’ for them.

“Will get bored. [People] will say, on daily basis-everyday what? Tablet-tablet! [/angry expression/]”. [Urban SBSW, 01-10-56, Pune, MH].

‘Boring’ was the state of mind that was constantly voiced by other stakeholders as something repetitive which is not acceptable. To prevent boredom with oral PrEP, other suggestions were that of intermittent dose or coitus dependent regimen that will not bind them to daily regimen:

“I have to eat [/take PrEP/] regularly, then people will be bored. This means they will miss out or become bored. So is it anything like this in that tablets, that the tablets are taken  only while during sex or like today suppose I want to do or want after one or two hours…. So instead of taking regularly, if the tablets [/PrEP/] will come like before the sex only, maybe half an hour, before 15-20 minutes should be taken then. There will not be any problem at all in that” [Key informant_Urban bisexual man, 01-30-11, Pune, MH].

*Typological barriers*: The condition for non-use of oral PrEP would also depend on the typologies of the FSWs. Over half of the BBSWs (68%) and almost a quarter of SBSWs (33%) cautioned that there would be differential adherence to oral PrEP among FSWs belonging to various typologies. The situations and context are different for different typologies of FSWs. The typological barriers emerged because of the following issues: (1) anticipated cost of the product: affordability, (2) lack of privacy to take and store daily dose and, (3) adherence challenges due to life style, working hours and working space. For example, a home-based sex worker would have probably no privacy to continue with the oral PrEP while FSWs, who were on daily-wages, would not be able to afford it daily because of cost as well as disrupted meal timings:

“Taking it daily in a family will not be easy. There is a possibility to forget due to situations like quarrels, any problem, and any work tension. If we become tense due to any problem, we might forget and then later worry about it” [Urban BBSW, 05-10-27, Chennai, TN].

“…majority of people are on daily wages [/no time or privacy; cost/], so it is difficult to take the tablet [/PrEP/] regularly” [Rural SBSW, 06-10-43, Vellore, TN].

Affordability emerged as a major challenge for adherence cited in case of adherence problems in treatment related to non-communicable diseases and even prevention medicines. Majority of the FSWs demanded free or subsidized oral PrEP:

“It will be good if the government provides some concession or else free of cost on medicine for the people like us [/FSWs/] so that it will be very much helpful to take regularly” [Rural BBSW, 06-10-44, Vellore, TN].

### Adherence to oral PrEP: facilitators of adherence

Adherence facilitators were very specific to the profession of FSWs. Their work required long-acting PrEP. Their work also required physical beauty/ attributes that they perceived that made them more attractive. Following two facilitators of adherence emerged: (1) long-acting PrEP, (2) monitoring.

*Long-acting PrEP:* This emerged as the only facilitator for adherence from rural as well as urban FSWs:

“It’s good if the tablet comes for 3 months once or twice but it should not be [/meant to be/] taken daily” [Rural SBSW, 06-10-43, Vellore, TN].

“The tablet [/PrEP/] can be made such that it can be taken weekly once or monthly once instead of daily. It will be better for people to take it consistently” [Urban SBSW, 05-10-21, Chennai, TN].

“Once we take for 2-4 months, if it works it is ok, or for once it works for one day it is ok.” [Urban SBSW, 03-10-31, Belagavi, KT]

“It will be good if the tablet [/PrEP/] can be taken once in six months or yearly once, I feel difficult to use it daily” [Urban FGD, FSW-2, Chennai, TN].

An FSW took the example of national guidelines for HIV testing for key populations to justify a long-acting PrEP as follows:

“Hmm! how our test [/HIV testing/] happens monthly or two months or three monthly? The same way women should get an injection like that … so then she won’t get bored” [Urban SBSW, 01-10-13, Pune, MH].

In the context of adherence, the FSWs put forth their fear of missing dose and the consequent repercussions of acquiring HIV. They tried to extrapolate from their past experiences with contraceptive pills where missed doses had led to conception. ‘What happens if a dose is missed in case of unprotected sex’? This question was raised in several contexts particularly when these women were not confident of optimal adherence. A need to understand the impact of ‘missed’ dosage emerged as responded by the FSW in Chennai:

“If we see the contraceptive pills, we are taking it continuously for 30 days. If we leave it even for one day, we may conceive. But, for this [/oral PrEP/], if we take this continuously and leave it for one day, we should not get any effects [/HIV/]. If it is like that, then it is OK, or else it is difficult to take it continuously” [Urban BBSW, 05-10-27, Chennai, TN].

Desire for long acting injectable PrEP emerged.

“Injection, if taken once, then there will be no tension up to one-one and a half year” [Urban SBSW, 01-10-56, Pune, MH].

*Monitoring:* FSWs emphasized that community is error-prone when it comes to behaviors, especially health behaviors. Hence a simple logic was posed by them—‘no one gets punished for having a delay in meals’ and similar principle should apply to oral PrEP. Concern for adherence followed logical exploration about the monitoring needs. FSWs suggested the role of NGOs in retention and adherence to overcome the problem of missing dose:

“Similarly they may not miss the tablet. Eventually, someone must follow them from the NGO to [/remind them to/] take the tablet regularly” [Rural SBSW, 06-10-43, Vellore, TN].

## Discussion

FSWs were able to dwell upon the risk and uncertainties of their situation and use of oral PrEP. They discussed the effective evaluation of many alternatives and thus demand for oral PrEP became evident among FSWs both in the rural and urban settings in India. FSWs are treated as non-independent entities: (1) they can neither refuse clients nor regular partners, (2) they are in the broad category of the key population but have their unique ramifications of risks. The fear of ‘rape’ was prevalent across urban–rural setting and as well as among street-based or brothel-based FSW. Brothel keepers were empathetic to the vulnerabilities in terms of coercion and rape of FSWs. The role of brothels in empowering sex workers in India have been well documented [21]. Therefore, the empathy of brothel keepers and the trust of FSWs for them in this study can be utilized by involving brothels in oral PrEP rollout. Oral PrEP is seen by the FSWs as protection in complete privacy. Although, FSWs reportedly did not want HCP initiated PrEP, yet initially HCPs would need to be involved for monitoring. It would require planned advocacy, explaining the need for HCP to assess the safety monitoring of oral PrEP.

The need for oral PrEP emerges from reported vulnerabilities of coercive sex, hitherto, not discussed much in sex work settings. Positive prevention or male condom usage, in either case, the control lies with the ‘index case/ man’ rather than the ‘woman’ who might acquire HIV infection from the client or the male partner. Thus, women who sell sex, might need self-controlled new HIV prevention options to protect themselves. A modeling study at Kenya and Ukraine estimates that a reduction of approximately 25% in HIV infections among sex workers may be achieved when physical or sexual violence is reduced [22]. In our study FSWs from both the rural and urban settings professed their inability to negotiate male condom use because they were duped into coercive or multi-partner sex. Addressing violence would require structural intervention and program. However, to prevent unsafe sex in such scenarios oral PrEP or any other  PrEP, would provide the much needed protection to a sex worker leading to reduction in HIV infections in this key population. The ease and independence of the use of oral PrEP have been discussed by all participants. An alternative to male condoms was a demand among the FSWs. Our study shows that oral PrEP could be more empowering than condoms as oral PrEP in sex workers’ words ‘needs to be swallowed’ only. Hence, oral PrEP could be an important tool of prevention for this key vulnerable population.

Although the FSWs have responded to the HIV prevention program in India [23–26], yet in this study, the concept of prevention seemed to be difficult to practice and sustain. Our study found unique opportunities and challenges around PrEP usage among these different groups of FSWs. The compulsion to consume alcohol in their profession made FSWs realize their vulnerability and difficulty in adhering to PrEP. In an inebriated state of mind, FSWs acknowledged that they could not think about their protection. In that situation, ‘ease of using a pill’ rather than any other prevention product seemed to be a highly acceptable prevention option empowering them to be prepared before they became intoxicated and vulnerable. They state that it might be difficult to think about daily PrEP when intoxicated. The locus of control was aptly perceived to lie with the FSW and nothing was easier than swallowing a pill; hence a strong willingness to use oral PrEP as HIV prevention option among FSWs emerges from this study. Unlike other studies where long acting injectable PrEP was preferred [27], in our study, FSWs wanted ‘pill’ on which they could have control unlike injectables which were perceived as health care provider driven. However, long acting injectable PrEP were desirable for obvious reasons of not going to HCPs frequently. FSWs from both urban and rural settings voiced their fear of stigma and tablet was an acceptable formulation both to circumvent stigma; can be used independently and therefore help maintain privacy. FSWs reported being more comfortable with NGO/ CBOs functionaries as compared to clinicians with whom they felt awkward or stigmatized. This addresses the problem of the potential burden on HCPs because community-based implementers were reportedly more acceptable. Along with brothel keepers, grassroots level workers from the community (Anganwadi workers) were the acceptable implementer option and they can share the task during oral PrEP implementation. This can bring about better community acceptance and might ensure adherence to PrEP.

Oral PrEP was perceived as a tool for economic enhancement among the FSWs. Other studies have also shown that FSW might forgo condom use for earning more [28]. Most of the participants were concerned about protection from HIV; only 1–2 fleetingly referred to need for STI prevention. Therefore, risk compensation remains a concern and we know that condoms need to be used for pregnancy and sexually transmitted infections prevention. FSWs should be reminded strongly that oral PrEP would not protect them from other STIs or pregnancy. In mathematical modeling, it has already been shown that the impact of PrEP may be strongly diminished or even reversed by behavioral disinhibition, especially in scenarios with low coverage and low effectiveness [29]. Thus, oral PrEP as an addition and not as substitution should be offered in the Indian setting. Roll out with a strong statutory warning that it might protect against HIV but additional prevention options would be required for STI/ pregnancy prevention should be implemented. Roll out of oral PrEP would require policymakers to recommend such legislations.

Issues of non-acceptability of oral PrEP among regular condom users also emerged which can be solved by bringing oral PrEP as an addition to the prevention option without undermining existing prevention programs. However, it is also to be noted that switching over from condom to PrEP and vice versa might not reverse the effects of PrEP even if condom use reduces by 50% among sex workers [30].

Poverty breeds compromises and in this study, the examples of compromises were rampant use of alcohol and substance along with the addition of condom less sex among FSWs. This has also been reported in another study conducted in India [31]. Zhang et al. (2013) stated that the compulsion to consume alcohol is a typical vulnerability of low paid sex workers and in their study, they were generally street-based sex workers [32]. By this definition, in our study, rural FSWs draw attention to their lowest level of safety and high episodes of violence and coercion ranking them as the lowest in the hierarchy of sex workers. Rural FSWs also pointed out that being daily wage earner and related this to their inability to adhere to a daily dosage of PrEP. It not only indicates affordability issues but also adherence could be a concern for a daily regimen. Concerns about affordability if sex worker is payer were raised, but it was suggested by the sex workers that it be provided at subsidized price or free as part of an HIV prevention intervention. The interventions would be a challenge in a rural setting but an urgent need for prevention options is critical. The rural FSWs wanted the involvement of government-run ART centers for dispensing and follow-up which was very different from what urban FSWs wanted who preferred NGOs/ CBOs for PrEP distribution.

Concerns for side effects have been voiced worldwide [33]. The systemic side effects could be a deterrent to oral PrEP usage among FSWs but might be tolerated with medical intervention and counseling especially the self-limiting ones [34]. The FSWs in this study appeared very cautious in sharing their preference for oral PrEP. Evidence of intrinsic fear of side effects emerged repeatedly; almost all of them put forth the condition that the product should be tested first as none of them had ever used oral PrEP. A need for a demonstration project also emerged as FSWs wanted to ‘see and use’ the product before forming any concrete opinion on the optimal usage of oral PrEP. Several side effects which were the symptoms of Reproductive Tract Infections (RTIs) were openly associated with any medical product being used by them. The demonstration project might  also address the challenge of such mythical side effects that were cited by FSWs. Counseling about possible side effects and symptoms of RTIs/STIs should be a part of a PrEP rollout program.

Adherence remains a strong prescription both for HIV prevention and treatment. Three of the five influencers stated by Parkin [17] tend to influence the decision to take daily oral PrEP. The personality of not liking repetitive things (viz. daily regimen), personal values such as faith upon regular partner and having alcohol as social and occupational norms were constraints emerging for the use of daily oral PrEP among this population. The personal value also emerged, when the apprehension about side effects on their physical attributes that are especially valued in the profession of this key population emerged. The side effects on the physical attribute i.e. the beauty and reproductive health were not at all acceptable in place of any kind of protection/ prevention. FSWs would need to be assured about the safety of physical attributes and the reproductive system. The concern for the protection of beauty and reproductive parts provides a key message to the researchers, drug developers and pharmaceuticals that they must focus their attention on the side effects especially on body fat changes, side effects to the uterus or other reproductive parts. The occurrence of such side effects would spell the failure of oral PrEP rollout among the FSW population. FSWs were also concerned about the interaction between alcohol and oral PrEP. In PrEP trials, adherence emerged as one of the critical factors influencing effectiveness [35]. Adhering to the daily regimen was one of the most difficult problems stated by the FSWs in this study; especially in the wake of their routine alcohol/ recreational drug use. Alcohol and recreation as barriers to adherence to PrEP were reported elsewhere also [28]. The effectiveness of PrEP is directly proportional to adherence and adherence, as expected, remained a prominent challenge owing to alcohol, boredom, and fear of side effects. Messages clarifying that there would not be oral PrEP-alcohol interaction was a demand that needs to be fulfilled if optimal adherence is expected. Alongside it is also important to emphasize the messages that there would be an effect of alcohol on their behaviors that would have an impact on adherence.

As observed in large clinical trials [36], stigma about ART was a barrier for both acceptance and adherence to oral PrEP. ART, which is associated with HIV treatment, led to the fear of stigma and being labeled as HIV infected. In other studies too, regular pill taking of oral PrEP, alcohol consumption and irregular lifestyles and fear of being perceived as HIV positive has been documented in other studies too [28, 34]. Since oral PrEP would be an ART, it posed a barrier to acceptance and adherence to oral PrEP. FSWs’ concern for adherence emerged from their experience with family planning. The expectation was that oral PrEP default should be more forgiving i.e. one event of non-adherence should not lead to HIV acquisition. Hence emerges the need for products with longer half-life to achieve a target steady-state level more quickly [37] especially in the populations where adherence is bound to be sub-optimal. FSWs’ concerns regarding adherence of daily dosage is a critical issue. The unique feature of the FSW community appears to be their affinity for continuous change. They feared boredom and anything ‘routine’ was boring. ‘Boredom’ is known to breed sensation-seeking behavior [38] which perhaps explains an FSW's risky and sometimes pompous lifestyle. Easily bored people are discontent, at higher risk of depression, anxiety, drug addiction, alcoholism, etc. Therefore, in due course, this trait is bound to influence acceptability and adherence to prevention products in this population. Repetition not only made them wary of the long term side effects of oral PrEP, but they also felt that it compromised their ability to adhere to the daily regimen of oral PrEP. Hence, there was resistance to ‘daily’ PrEP. Again, long-acting HIV prevention regimens need to be pursued.

Stigma encompasses every aspect of FSWs’ life. To fight stigma, FSWs keep struggling and a critical situation is when she voluntarily refuses to use prevention methods. The dynamics of intimate relationships among FSWs and their regular partners show the non-efficacy of FSWs to use HIV prevention options. In a study among sex workers in Mexico, Robertson et al. suggested modifying the HIV risk framework which only focuses on constructs of disease and risk [39]. As in this Mexican study, FSWs in our study also ascribed a different meaning to their relationship with regular partners which needs to be considered for implementing oral PrEP rollout among FSWs in India. Psychological empowerment might overcome the adverse effects of stigma and exclusion among FSWs [40].

In line with another study [13], FSWs in this study also pointed towards stigmatizing health care system. Easy accessibility at pharmacies or involvement of CBOs or grass root workers from health system for dispensing was preferred and an aversion for health care provider mediated oral PrEP emerged. Oral PrEP seems to be more empowering because of the non-intervention of health care providers, unlike Kenyan FSWs who preferred injectable PrEP [28]. Our study emphasizes a need to clarify to the FSW population that initiation of oral PrEP and subsequent monitoring of side effects and adherence by skilled medical staff is critical for at least the initial few months. The expectation that oral PrEP will be freely available needs to be thwarted as early as possible. A peer-led approach for dispensing of PrEP in conjunction with formal health care settings is recommended. The ART retention model of peer involvement existing in India can be adapted for the oral PrEP program.

The social marketing model for oral PrEP delivery emerges. Program may need skilled trained resource who could help to achieve social change in this key population (empowerment esp. with various types of partners), promote and raise awareness about HIV prevention (Peer led PrEP, condoms and other prevention options awareness/ distribution), and induce changes in behavior (alcoholism, adherence). In rural areas, confidentiality was a larger concern and preference was for delivery through ART centers while in urban areas preference was for NGOs. This issue of dispensing needs to be explored. According to a UNAIDS meeting report (2014), the introduction of PrEP to sex workers should be integrated within the secure provision of comprehensive sexual health care, including HIV treatment. It also recommended widespread monitored access to PrEP is necessary to reduce informal and unregulated use [41]. In today’s scenario, Differential Service Delivery Model for PrEP delivery needs to be urgently explored in view of COVID 19 pandemic experiences.

An optimism emerged around PrEP but it is to be noted that FSWs have been placed in a wide bracket of people at risk of HIV. FSWs questioned the need for prevention in the absence of disease or symptoms indicating their right to exercise to make sexual health decisions on their own. This is also an expectation to define ‘substantial risk for oral PrEP indication’ like it is defined in case of Men having Sex with Men and transgender people. Their suggestions that oral PrEP should be ‘used first’ before they can give their preference, perhaps point out the gap such as lack of evidence of how would oral PrEP specifically impact them in their own socio-cultural and occupational setting in the long run. To improve effectiveness in real-world settings, more research is needed on feasibility of using PrEP in countries like India and the contextual factors surrounding women’s personal experiences with PrEP [42]. It does not exonerate the country from conducting a demonstration project among female sex workers. In the larger interest, a higher-order structural intervention is needed to make community members realize the goal of public health which is through prevention.

### Study limitations

The limitations is that data is not very current; however, since India is still preparing to roll out PrEP program, this study informs the program. We have not explored extensively about STIs and risk perceptions for STIs and HIV.

## Conclusion

Oral PrEP was acceptable to FSWs in both urban and rural settings and among BBSWs and SBSWs. Overall, condom-less sex was either, voluntary- to please regular partner/ spouse/ rich client, or it was forced and coercive. Rural FSWs were more vulnerable and they require contextually tailored focus for empowerment and HIV prevention. HCP intervention for PrEP was not preferred by the FSWs. Therefore either ‘pill’ that they could take themselves or long acting injectable PrEP which required minimal intervention from HCP were emerging need. Desire for event dependent PrEP pills also emerged. Awareness for initial medical monitoring is a gap that needs to be taken into cognition by the program managers. Side effects affecting physical beauty and reproductive organs could pose strong barriers to PrEP use. Social marketing principles should be employed to raise awareness and bring behavior changes to boost adherence to PrEP. To formulate a PrEP package in India, the broader HIV/AIDS-related national policies and programs may need to evolve to increase the availability, acceptability, and utilization of oral PrEP among the most vulnerable populations. Advocacy for long acting/ event dependent PrEP is recommended.

## Data Availability

Data will be available at the NARI’s centralized data repository system and is available on request to director@nariindia.org.

## Abbreviations

PrEP: Pre-exposure prophylaxis
FSW: Female sex workers
BBSW: Brothel based sex worker
SBSW: Street based sex worker
NGO: Non-governmental Organization
CBO: Community Based Organization
